# Early Fluid Resuscitation and High Volume Hemofiltration Decrease Septic Shock Progression in Swine

**DOI:** 10.1155/2015/181845

**Published:** 2015-10-12

**Authors:** Ping Zhao, Ruiqiang Zheng, Lu Xue, Min Zhang, Xiaoyan Wu

**Affiliations:** ^1^Intensive Care Unit, Subei People's Hospital of Jiangsu Province & Clinical Medical School of Yangzhou University, Yangzhou, Jiangsu 225001, China; ^2^Intensive Care Unit, Wujin People's Hospital & Clinical Medical School of Jiangsu University, Zhenjiang, Jiangsu 213017, China; ^3^Intensive Care Unit, Taizhou People's Hospital of Jiangsu Province & Clinical Medical School of Yangzhou University, Yangzhou, Jiangsu 225300, China

## Abstract

This study aimed to assess the effects of early fluid resuscitation (EFR) combined with high volume hemofiltration (HVHF) on the cardiopulmonary function and removal of inflammatory mediators in a septic shock swine model. Eighteen swine were randomized into three groups: control (*n* = 6) (extracorporeal circulating blood only), continuous renal replacement therapy (CRRT) (*n* = 6; ultrafiltration volume = 25 mL/Kg/h), and HVHF (*n* = 6; ultrafiltration volume = 85 mL/Kg/h). The septic shock model was established by intravenous infusion of lipopolysaccharides (50 *µ*g/kg/h). Hemodynamic parameters (arterial pressure, heart rate, cardiac output, stroke volume variability, left ventricular contractility, systemic vascular resistance, and central venous pressure), vasoactive drug parameters (dose and time of norepinephrine and hourly fluid intake), pulmonary function (partial oxygen pressure and vascular permeability), and cytokines (interleukin-6 and interleukin-10) were observed. Treatment resulted in significant changes at 4–6 h. HVHF was beneficial, as shown by the dose of vasoactive drugs, fluid intake volume, left ventricular contractility index, and partial oxygen pressure. Both CRRT and HVHF groups showed improved removal of inflammatory mediators compared with controls. In conclusion, EFR combined with HVHF improved septic shock in this swine model. The combination decreased shock progression, reduced the need for vasoactive drugs, and alleviated the damage to cardiopulmonary functions.

## 1. Introduction

Severe infections are a major cause of death in intensive care units (ICU) [1]. The in-hospital mortality rate due to severe infections is 27%, and approximately 54% of this mortality is due to septic shock [2] because of the direct relationship between sepsis, systemic inflammatory response syndrome (SIRS), and compensatory anti-inflammatory response syndrome (CARS) [3].

While it may be beneficial to fight the systemic infection, it may also be deleterious, leading to multiorgan failure and death [4–7]. Moreover, inflammatory mediators with direct cytotoxic effects, hypersecretion of proinflammatory mediators, and prolonged release of anti-inflammatory mediators can all directly lead to tissue and organ injuries [7]. Therefore, in the early phase of sepsis, hemofiltration can attenuate and stop the inflammatory cascade, thus alleviating cell and tissue damage and reducing the mortality due to multiple-organ failure syndrome. High-volume hemofiltration (HVHF) can be performed by increasing the amount of replacement fluid, thus improving the removal of soluble macromolecules.

Nevertheless, precise HVHF definition and ultrafiltration flow rate and selection and treatment opportunity and timing have not been fully elucidated [8]. According to several clinical studies, the highest HVHF rate is 200 mL/kg/h [9], while the lowest rate is approximately 40–60 mL/kg/h [10, 11]. However, most of these studies are small-scale single-center trials, and results on hemodynamics improvement and mortality are not consistent. In the IVOIRE (hIgh VOlume in Intensive caRE) study [12], patients with severe infection and acute kidney injury (AKI) were treated with CRRT and randomly assigned to hemofiltration rates of 35 mL/kg/h or 70 mL/kg/h. However, the IVOIRE study did not show any prognostic advantage for the 70 mL/kg/h hemofiltration rate [13]. In contrast, results indicate that patients with AKI should be treated with CRRT in the early phase of RIFLE criteria. Though most clinical studies [14–16] focused on patients with AKI or acute renal failure (ARF), the necessity of using hemofiltration at the onset of ARF to treat septic shock is still controversial. Early HVHF treatment of patients with septic shock with or without organ dysfunction/failure has been investigated [17, 18].

Likewise, in most animal studies [19–21], the effects of HVHF were shown to be beneficial in many aspects. Importantly, early fluid resuscitation (EFR) was not used when creating the animal models. As a result, these experiments suffer from significantly inadequate tissue perfusion. Thus, in the present study, we conducted EFR immediately after the establishment of a septic shock model in swine, and we used the pulse contour cardiac output (PiCCO) monitoring system to observe the therapeutic effects of EFR combined with HVHF in this model.

## 2. Materials and Methods

### 2.1. Animals and Ethical Considerations

Animals used in the study were healthy local swine (kindly provided by the Veterinary Department of YangZhou University), aged 9-10 weeks, weighing 23–25 kg, and of both genders. All experiments were performed at the Department of Medicine of YangZhou University, which possesses all necessary authorizations to perform animal experiments. All individuals performing animal experiments were properly qualified, and the study was approved by the local ethics committee of the university.

### 2.2. Anesthesia and Catheter Insertion

Swine were intramuscularly injected with 150 mg of ketamine. Ten minutes later, 2 mg of midazolam was intravenously injected into the ear margin. A 5-lead ECG was immediately used to monitor cardiac electrical activity, and an Evita 4 ventilator (Dräger, Lubeck, Germany) was used to assist breathing using orotracheal intubation (model number 5-5.5). The ventilator was set to the intermittent positive-pressure ventilation (IPPV) mode, and the parameters were tidal volume (TV) 10 mL/kg, frequency (*F*) 16 times/min, and FiO_2_ 40%. Midazolam and vecuronium were continuously intravenously injected (0.06 mg/kg/h and 0.07 mg/kg/h, resp.) to maintain anesthesia. We next inserted PiCCO (Pulsion Medical Systems, Feldkirchen, Germany) arterial catheter into the right femoral artery. We inserted a dual-chamber blood filter catheter (8F, China) into the right jugular vein. A dual-lumen central venous catheter (ARROW Dual-Lumen, China) was then inserted into the right forelimb vein. The main chamber was connected to a pressure transducer to monitor central venous pressure and was installed with a PiCCO catheter temperature sensor. The other chamber was used for fluid infusion. All systems were allowed to stabilize for 30 minutes. Next, 3 mL of saline (<8°C) was injected into the main chamber via the dual-lumen central venous catheter for thermodilution measurement; the injection was repeated 3 times. We then measured several baseline values including heart rate (HR), mean arterial pressure (MAP), global end dilution volume (GEDV), stroke volume (SV), stroke volume variability (SVV), cardiac output (CO), extravascular lung water (EVLW), central venous pressure (CVP), systemic vascular resistance (SVR), and left ventricular contractility index (dPmax). We also recorded the ventilatory index and collected arterial blood for blood gas analysis using a GEM Premier 3000 (Instrumentation Laboratory, Bedford, MA, USA).

### 2.3. Experimental Design

This study was divided into three phases. The first phase was the establishment of the septic shock model. After recording the baseline MAP value, endotoxin (0111: B4, Sigma, St. Louis, MO, USA) was continuously injected (50 *µ*g/kg/h) via the central vein under full anesthesia, analgesia, and comprehensive monitoring. The threshold of successful model establishment was set as a 30% reduction in the MAP value.

The second phase was EFR. Once the septic shock model was established, EFR using crystalloid (compound sodium chloride injection, Shijiazhuang Number 4 Pharmaceutical Co., Ltd., China) was immediately administered via the central vein (15–20 mL/kg/h), and the resuscitation endpoint was defined as the PiCCO-monitored SVV value being decreased by no more than 10%, under full sedation. If the endpoint was not achieved, norepinephrine was given to maintain MAP at 90–100% of baseline. Resuscitation fluid volume and norepinephrine dose were recorded.

The third phase consisted of blood purification therapy. Swine were randomly divided into three groups: a control group (group 1), a CRRT group (group 2), and a HVHF group (group 3). Blood flow velocity was set at 100 mL/min in all groups. Group 1 was treated with extracorporeal blood circulation only. The predilution ultrafiltration rates of groups 2 and 3 were 25 mL/kg/h and 85 mL/kg/h, respectively. These rates were based on the Pardubice consensus definition [22, 23]. During treatment, fluid was supplemented according to the SVV index, at a rate of 6–10 mL/kg/h; the standard was set as SVV < 10%. Additionally, the dose of norepinephrine was adjusted to maintain baseline MAP levels.

### 2.4. Hemofiltration

A Prisma M60 hemofiltration column (Hospal, Lyon, France) and a PRISMA System hemofiltration machine (GAMBRO Renal Products, Medolla, Italy) were used. The replacement fluid contained 2000 mL of saline, 500 mL of 5% glucose solution, and 125 mL of 25% sodium bicarbonate. Zero balance, pH, and electrolyte levels were adjusted according to the results of blood gas analysis. Heparin (1500 U/h) was injected for anticoagulation. Body temperature was maintained using an insulation blanket.

### 2.5. Laboratory Tests

Arterial blood samples were collected for blood gas analysis; all samples were obtained from the same site. Samples were collected at baseline (i.e., upon establishment of the septic shock model) and at 1, 2, 3, 4, 5, and 6 h after treatment. Blood samples were centrifuged at 4000 rpm for 15 min, and serum was obtained to measure levels of inflammatory mediators.

### 2.6. Statistical Analysis

SPSS 17.0 (IBM, Armonk, NY, USA) was used for data handling and analyses. Normally distributed continuous data are presented as mean ± SD and were analyzed using ANOVA and the LSD post hoc test. Nonnormally distributed continuous data are presented as median (interquartile) and were analyzed using the Mann-Whitney *U* test. Categorical data are presented as frequencies and were compared using Fisher's exact test. SPSS 17.0 (IBM, Armonk, NY, USA) was used for data handling and analyses. Statistical significance was defined as *P* < 0.05.

## 3. Results

### 3.1. Successful Establishment of a Swine Septic Shock Model

Eighteen septic shock swine models were induced. One swine (#6 from the control group) had difficulties in maintaining cardiopulmonary functions after 3 hours of treatment in the third phase and died from arrhythmias.

**Figure 1:**
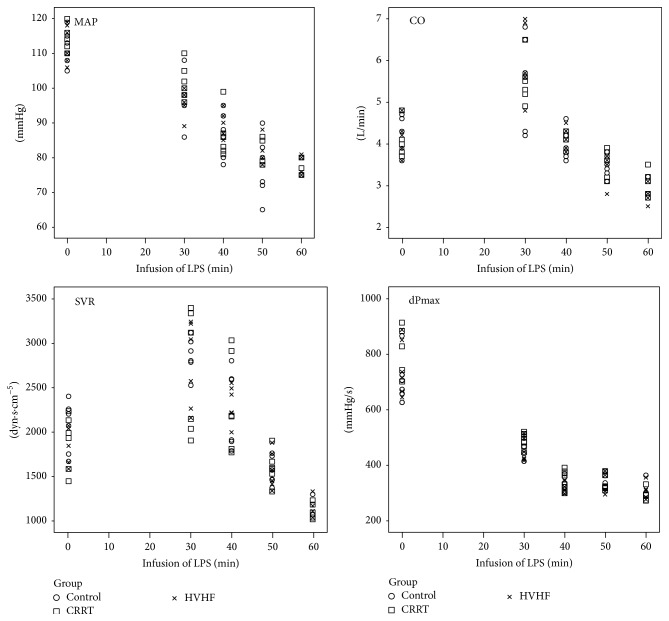
Hemodynamic variations observed during the establishment of the swine septic shock model. MAP: mean arterial pressure, CO: cardiac output, SVR: systemic vascular resistance, and dPmax: left ventricular contractility index. There was no significant difference between the three groups at any time (*P* > 0.05).

All animal models were successfully obtained within 45 minutes and 1 hour of treatment and showed typical signs of septic shock upon establishment including significant decreases in MAP, CO, and SVR and progressive increases in HR and BT (Figure 1 and Table 1). Obvious SIRS occurred in all animals. EFR was performed, and we found that SVV was maintained between 8 and 10% and MAP was maintained at 90–100% of baseline. At the time of successful resuscitation, there were significant increases in CO and dPmax and significant decreases in HR compared with baseline (Table 2), indicating that the typical septic shock model (high CO and low SVR) had been successfully established. There were no significant differences between the three groups before the treatment phase.

### 3.2. HVHF Improves Cardiac Function in the Septic Shock Model

**Figure 2:**
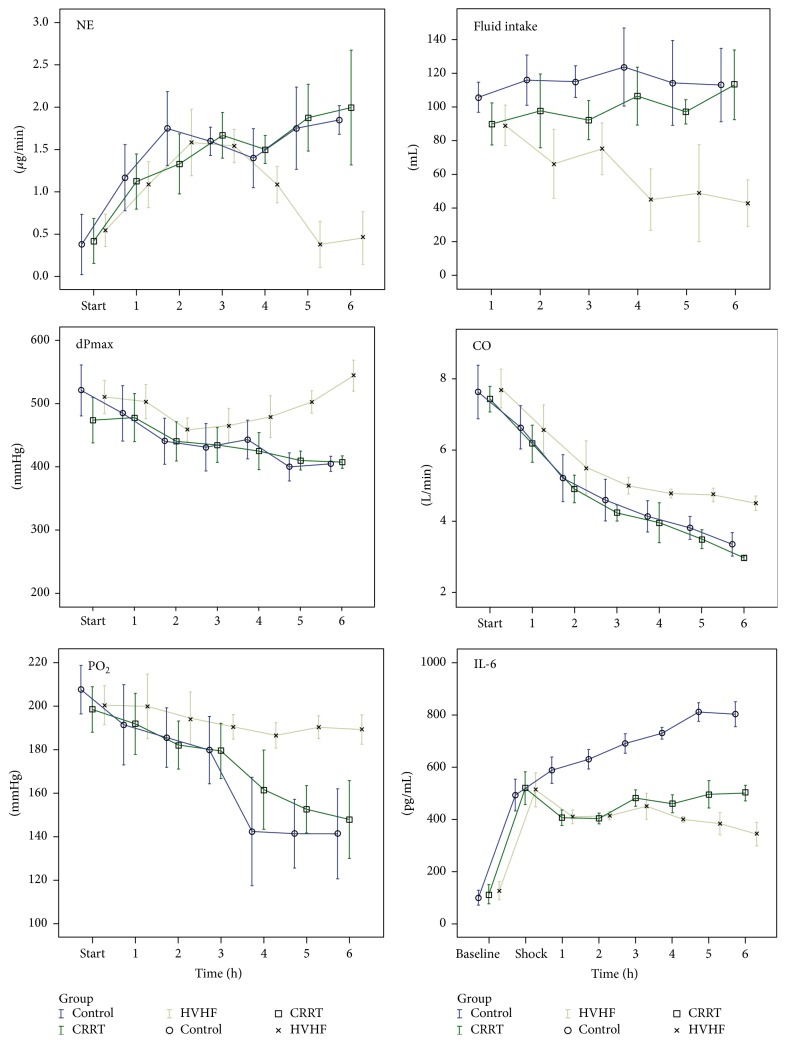
Parameter variations observed in different groups during treatment. CO: cardiac output, dPmax: left ventricular contractility index, NE: highest norepinephrine dose, fluid intake: fluid intake volume per hour, PO_2_: arterial oxygen pressure, and IL-6: serum interleukin level.

Compared with levels at successful resuscitation, CO levels of all three groups were decreased. Data at 6 h showed that the HVHF group had the smallest decrease and that CO was higher compared with baseline. The CO of the other two groups was significantly lower compared with baseline. The dPmax of the HVHF group at 4, 5, and 6 h was significantly higher compared with the other two groups; however, baseline levels were not reached by 6 h (Table 2, Figure 2).

### 3.3. Norepinephrine Dose and Fluid Intake Are Decreased in the HVHF Group

During the treatment phase in all three groups, the dose of norepinephrine was gradually increased during the first 3 hours before successful resuscitation. Data at 4, 5, and 6 h showed that the norepinephrine dose was lower in the HVHF group and that it was significantly lower compared with the CRRT and control groups (*P* < 0.05). There was no significant difference between the CRRT and control groups (*P* > 0.05). Fluid intake of the HVHF group was significantly lower compared with the other two groups during the third phase, and fluid intake of the CRRT group was significantly lower than that in the control group (*P* < 0.05). However, this difference was not significant at 6 h (*P* > 0.05) (Table 2, Figure 2).

### 3.4. HVHF Improves Partial Oxygen Pressure and Preserves Pulmonary Vascular Permeability in the Septic Shock Model

Compared with baseline, PO_2_ was decreased in all three groups. Moreover, there was a significant difference (*P* < 0.005) between the HVHF group and the two other groups at 4, 5, and 6 h. PO_2_ in the CRRT group was significantly higher than that in the control group (*P* < 0.05). For PVPI at 3, 4, 5, and 6 h, there was a significant difference (*P* < 0.05) between the HVHF group and the two other groups. There was a significant difference (*P* < 0.05) in PVPI between the CRRT and control groups at 4, 5, and 6 h (Table 2, Figure 2).

### 3.5. HVHF Improves the Removal of Inflammatory Mediators Associated with Septic Shock

Upon establishment of the septic shock model, IL-6 and IL-10 levels increased dramatically. The HVHF and CRRT groups showed an effective removal of inflammatory mediators (*P* < 0.05 versus controls). Although the decreases in the HVHF group were more obvious, the levels of inflammatory mediators did not decrease to baseline levels by 6 h (Table 3, Figure 2).

## 4. Discussion

The aim of the present study was to assess the therapeutic effects of EFR combined with HVHF on cardiopulmonary functions and removal of inflammatory mediators in a septic shock swine model. Results showed that EFR during HVHF was beneficial for the treatment of septic shock. This approach can reduce the levels of inflammatory mediators by increasing their removal, improve cardiopulmonary function, and decrease the dose and maintenance time of vasoactive drugs. Severe infection or septic shock will lead to hypoperfusion and organ failure, which is largely due to an imbalance in inflammatory mediators. Research has shown that CRRT is able to remove inflammatory mediators and, thus, attenuate the inflammatory cascade. As such, it has been used in the treatment of critically ill patients with sepsis and multiorgan dysfunction syndrome.

With increased inflammation, MAP and SVR decrease and vascular permeability increases. Effective EFR, performed within this time window, contributes to an adequate volume status, and the increase of extravascular lung fluids would affect pulmonary oxygenation and cardiac function. Results showed that the effects of HVHF on cardiopulmonary function in the swine septic shock model occurred 4 to 6 hours after hemofiltration and were mainly manifested by CO, dPmax, PO_2_, and PVPI. In addition, in the CRRT and HVHF groups, CO and dPmax were close to baseline levels by 6 h after treatment. Additionally, fluid intake was significantly reduced and pulmonary vascular permeability and oxygenation were improved. Though there are some similarities between the results presented here and previously published work, we observed that EFR combined with HVHF significantly reduced organ damage.

For HVHF dose selection, both published studies and our own experience were taken into consideration. After reviewing recent small-scale clinical studies, the IVOIRE study [12, 13], and the Pardubice consensus definition [22, 23], ultrafiltration rates of 25 mL/kg/h and 85 mL/kg/h were selected, with the goal of being applicable to future clinical studies and to avoid known drawbacks in HVHF treatment [24, 25].

Bellomo et al. [26] have shown how different ultrafiltration volumes influence the dose of vasoactive drugs for the treatment of septic shock. They also showed that HVHF could significantly decrease the use of norepinephrine and decrease hypotension development. Yekebas et al. [21] compared the effects of different filtration volumes and column replacement in a pancreatitis swine model. Their results suggested that HVHF and column replacement were advantageous in improving MAP and in prolonging animals' survival. However, in most animal studies, treatments were performed immediately after model establishment; importantly, this differs from clinical trials and is against the concept of administering EFR for septic shock [27].

It has been suggested that SVV monitoring should be implemented because it better reflects the patient's sensitivity to liquid volume and more accurately monitors liquid volume [28]. Therefore, the PiCCO system was used in the present study, and full EFR was performed immediately after establishing the septic shock model. In the present study, MAP and SVV levels were maintained and an adequate amount of effective circulating blood volume was kept to simulate a clinical trial environment. Blood gas analysis and PO_2_ measurements were combined to monitor hemodynamics and cardiopulmonary function.

Hemofiltration can improve hemodynamics and cardiopulmonary functions by removing inflammatory mediators including several myocardial depressant mediators [19]. Indeed, most hemofiltration membranes possess some adsorptive properties, allowing them to adsorb inflammatory mediators with higher molecular weights both onto the surface and into the bulk of the membrane (at least for modern membranes) [29]. Admittedly, we cannot ignore the efficiency of HVHF, since there was a significant decrease in inflammatory mediators using this approach. However, according to variations in the dose of vasoactive drugs and infusion volume, there was no significant difference in vasoactive drug use between the CRRT and control groups. Yekebas et al. [21] suggested that frequent column replacement is helpful to adsorb inflammatory mediators, and another study [30] suggested that this membrane adsorption is only temporary and that long-term effects could not be observed, which is a limitation of the present study.

The present study is not without limitations. In addition to not changing the membrane, the model used in the present study does not allow the evaluation of survival. Nevertheless, the IVOIRE trial reported that no prognostic advantage was observed between HVHF at 35 and that at 70 ml/kg/h [13]. However, in the IVOIRE trial, one-third of the dose was given in predilution mode, while the present study used a full-predilution mode. The use of a full-predilution mode is safer, with a lower likelihood of clotting, but the efficacy is reduced [4]. In addition, future studies should include a control group in which fluid resuscitation is delayed. Future studies should be designed to try to address this issue.

## 5. Conclusions

In a swine model of septic shock, EFR combined with HVHF treatment was beneficial to reduce damage to organs. This treatment could significantly inhibit shock progression, reduce the dose of vasoactive drugs, and alleviate damage to cardiopulmonary functions, all of which are considered to be associated with the removal of inflammatory mediators.

## Figures and Tables

**Table 1:** Hemodynamic parameters observed during animal model building.

Variable	Baseline	30 min	40 min	50 min	60 min	*P* ^*∗*^
MAP (mmHg)	113 ± 4.7	98 ± 5.9	87 ± 5.7	80 ± 6.2	78 ± 2.5	<0.005
HR (bpm)	109 ± 4.4	130 ± 3.7	136 ± 4.7	144 ± 6.5	149 ± 5.4	<0.005
CO (L/min)	4.1 ± 0.4	5.6 ± 0.8	4.0 ± 0.3^a^	3.4 ± 0.3	2.9 ± 0.3	<0.005
SVV (%)	9.1 ± 1.3	19.7 ± 1.7	24.2 ± 0.7	26.2 ± 0.6	26.8 ± 1.3	<0.005
dPmax (mmHg/s)	763 ± 98.9	467 ± 38.4	334 ± 29.0	344 ± 29.8	311 ± 33.2	<0.005
BT (°C)	38.8 ± 0.4	39.2 ± 0.4	39.4 ± 0.2	39.7 ± 0.3	40.1 ± 0.5	<0.005
SVR (dyn·s·cm^−5^)	1931 ± 274.5	2749 ± 485.6	2289 ± 400.5	1564 ± 176.6	1127 ± 104.3	<0.005
CVP (mmHg)	6.1 ± 2.1	—	—	—	3.9 ± 1.9	<0.005

MAP: mean arterial pressure; HR: heart rate; CO: cardiac output; SVV: stroke volume variability; dPmax: left ventricular contractility index; BT: blood temperature; SVR: systemic vascular resistance; CVP: central venous pressure.

^*∗*^ *P* < 0.005 and ^a^ *P* = 0.535 versus baseline.

**Table 2:** Parameters during treatment according to groups.

Variable/ group	Upon successful resuscitation	1 h	2 h	3 h	4 h	5 h	6 h
CO (L/min)							
Control	7.8 (7; 8.3)	6.7 (6.2; 7.1)	5.5 (4.5; 5.7)	4.5 (4.2; 5.1)	4.1 (3.9; 4.5)	3.8 (3.6; 4.0)	3.4 (3.1; 3.6)
CRRT	7.3 (7.2; 7.7)	6.4 (5.7; 6.6)	4.9 (4.6; 5.3)	4.3 (4.1; 4.4)	4.0 (3.5; 4.4)	3.6 (3.2; 3.7)	3.0 (3; 3.1)
HVHF	7.6 (7.2; 8.3)	6.4 (6.1; 7.1)	5.4 (4.9; 6.1)	5.0 (4.8; 5.2)	4.8 (4.7; 4.9)^bc^	4.8 (4.7; 4.9)^bc^	4.6 (4.3; 4.7)^bc^
*P*	0.612	0.407	0.278	0.110	0.010	0.002	0.002
HR (bpm)							
Control	125 (119; 127)	128 (124; 131)	129 (121; 134)	131 (121; 133)	121 (119; 128)	119 (117; 129)	123 (122; 126)
CRRT	121 (119; 124)	128 (118; 132)	131 (122; 135)	127 (120; 132)	123 (120; 129)	131 (128; 133)	120 (119; 122)
HVHF	124 (120; 127)	130 (120; 134)	123 (120; 131)	122 (118; 124)	125 (122; 127)	121 (119; 134)	120 (118; 128)
*P*	0.494	0.787	0.579	0.224	0.659	0.132	0.202
dPmax (mmHg/s)							
Control	533 (481; 555)	485 (446; 517)	432 (410; 476)	414 (407; 464)	457 (417; 462)	399 (383; 418)	402 (398; 412)
CRRT	468 (443; 511)	478 (443; 510)	440 (409; 466)	437 (412; 458)	427 (397; 450)^a^	412 (398; 423)	411 (400; 414)
HVHF	500 (490; 540)	505 (480; 525)	459 (443; 476)	456 (449; 481)	479 (447; 512)^bc^	505 (485; 516)^bc^	546 (531; 565)^bc^
*P*	0.103	0.404	0.345	0.211	0.035	0.003	0.004
Infusion volume (mL)							
Control		107.0 (97.0; 112.5)	116.5 (106.3; 124.8)	112.0 (108.5; 123.0)	135.0 (103.5; 138.5)	108.0 (97.5; 134.0)	113.0 (98.5; 128.0)
CRRT		88.0 (79.3; 102.5)^a^	90.0 (85; 107.5)^a^	94.5 (82.5; 101.0)^a^	105.0 (92.5; 123.3)^a^	96.0 (93.0; 100.3)^a^	117.5 (92.5; 130.8)
HVHF		87.0 (81.3; 95.8)^bc^	71.0 (45.3; 83.8)^bc^	76.0 (58.8; 88.3)^bc^	46.0 (25.8; 59.5)^bc^	58.0 (27; 68.5)^bc^	40.5 (33.5; 52.3)^bc^
*P*		0.041	0.003	0.003	0.002	0.003	0.004^b^
Highest dose of norepinephrine (*μ*g/min)							
Control	0.4 (0; 0.8)	1.0 (0.9; 1.6)	2.0 (1.4; 2.0)	1.5 (1.5; 1.8)	1.5 (1.1; 1.6)	1.8 (1.4; 2.1)	1.8 (1.8; 2.0)
CRRT	0.5 (0.2; 0.6)	1.0 (0.9; 1.5)	1.3 (1.0; 1.8)	1.8 (1.4; 1.8)	1.5 (1.4; 1.6)	1.9 (1.5; 2.1)	1.9 (1.4; 2.6)
HVHF	0.5 (0.4; 0.8)	1.0 (0.9; 1.3)	1.8 (1.2; 1.8)	1.5 (1.4; 1.8)	1.1 (0.9; 1.3)^bc^	0.4 (0.2; 0.6)^bc^	0.5 (0.2; 0.8)^bc^
*P*	0.615	0.958	0.142	0.537	0.02	0.004	0.004
PO_2_ (mmHg)							
Control	210 (197; 218)	193 (176; 208)	184 (174; 200)	175 (143; 193)	130 (127; 165)	135 (132; 155)	136 (128; 159)
CRRT	196 (194; 203)	193 (181; 205)	179 (174; 195)	178 (171; 187)	165 (151; 175)^a^	155 (143; 162)^a^	146 (136; 166)^a^
HVHF	199 (194; 210)	208 (189; 209)	193 (184; 208)	191 (186; 195)	188 (182; 191)^bc^	190 (187; 194)^bc^	189 (184; 197)^bc^
*P*	0.194	0.306	0.204	0.144	0.002	0.002	0.004
PVPI							
Control	1.5 (1.3; 2.3)	1.6 (1.6; 2.3)	1.8 (1.6; 2.5)	3.0 (2.9; 3.5)	3.8 (3.4; 4.1)	4.0 (3.9; 4.4)	4.6 (4.4; 4.8)
CRRT	1.9 (1.8; 1.9)	1.9 (1.8; 2.1)	2.0 (1.9; 2.0)	2.9 (2.2; 3.1)	3.1 (2.7; 3.5)^a^	3.6 (3.5; 3.8)^a^	4.0 (3.8; 4.2)^a^
HVHF	2.0 (1.8; 2.2)	2.1 (1.7; 2.3)	2.1 (1.9; 2.6)	2.3 (2.1; 2.6)^bc^	2.4 (2.3; 2.6)^bc^	2.4 (2.3; 2.6)^bc^	2.4 (2.2; 2.8)^bc^
*P*	0.349	0.45	0.494	0.037	0.003	0.001	0.001

CRRT: continuous renal replacement therapy; HVHF: high volume hemofiltration; CO: cardiac output; HR: heart rate; dPmax: left ventricular contractility index, PVPI: pulmonary vascular permeability index; PO_2_: arterial oxygen pressure.

^a^ *P* < 0.05 between the CRRT and control groups, ^b^ *P* < 0.05 between the HVHF and control groups, ^c^ *P* < 0.05 between the CRRT and HVHF groups.

**Table 3:** Levels of serum inflammatory mediators.

Variable/group	Baseline	Upon successful resuscitation	1 h	2 h	3 h	4 h	5 h	6 h
IL-6 (pg/mL)								
Control	96 (75; 125)	475 (449; 549)	604 (533; 633)	627 (602; 668)	699 (663; 721)	734 (718; 750)	826 (785; 841)	785 (775; 848)
CRRT	99 (87; 152)	525 (475; 575)	403.5 (387; 434)^a^	400 (387; 424)^a^	479 (460; 517)^a^	465 (425; 495)^a^	501 (454; 540)^a^	493 (482; 536)^a^
HVHF	129 (92; 155)	507 (472; 559)	408.5 (390; 438)^b^	422 (404; 424)^bc^	447.5 (411; 506)^bc^	403 (394; 411)^bc^	394 (344; 421)^bc^	364 (294; 380)^bc^
*P*	0.330	0.581	0.003	0.003	0.004	0.001	0.001	0.001
IL-10 (pg/mL)								
Control	14 (11; 14)	68 (60; 78)	76 (72; 82)	91 (86; 97)	97 (91; 104)	98 (97; 106)	112 (101; 119)	106 (100; 127)
CRRT	16 (8; 18)	61 (56; 75)	67 (64; 74)^a^	51 (49; 52)^a^	50 (48; 55)^a^	45 (42; 46)^a^	41 (34; 46)^a^	37 (31; 39)^a^
HVHF	15 (12; 17)	75 (66; 80)	51 (46; 56)^bc^	41 (38; 48)^bc^	40 (34; 44)^bc^	31 (28; 33)^bc^	31 (27; 34)^bc^	27 (25; 30)^bc^
*P*	0.431	0.277	0.003	0.001	0.001	0.001	0.002	0.001

CRRT: continuous renal replacement therapy; HVHF: high volume hemofiltration.

^a^ *P* < 0.05 between the CRRT and control groups, ^b^ *P* < 0.05 between the HVHF and control groups, ^c^ *P* < 0.05 between the CRRT and HVHF groups.
